# Sensation Seeking, Non-contextual Decision Making, and Driving Abilities As Measured through a Moped Simulator

**DOI:** 10.3389/fpsyg.2017.02126

**Published:** 2017-12-11

**Authors:** Evelyn Gianfranchi, Mariaelena Tagliabue, Andrea Spoto, Giulio Vidotto

**Affiliations:** Department of General Psychology, University of Padua, Padua, Italy

**Keywords:** road safety, riding simulator, sensation seeking, decision making, novice road users

## Abstract

The general aim of the present study was to explore the relations between driving style (assessed through a moped riding simulator) and psychological variables such as sensation seeking and decision making. Because the influences of sensation seeking and decision making on driving styles have been studied separately in the literature, we have tried to investigate their mutual relations so as to include them in a more integrated framework. Participants rode the Honda Riding Trainer (HRT) simulator, filled in the Sensation Seeking Scale V (SSS V), and performed the Iowa Gambling Task (IGT). A cluster analysis of the HRT riding indexes identified three groups: Prudent, Imprudent, and Insecure riders. First, the results showed that Insecure males seek thrills and adventure less than both Prudent males and Insecure females, whereas Prudent females are less disinhibited than both Prudent males and Insecure females. Moreover, concerning the relations among SSS, decision making as measured by the IGT, and riding performance, high thrill and adventure seekers performed worse in the simulator only if they were also bad decision makers, indicating that these two traits jointly contribute to the quality of riding performance. From an applied perspective, these results also provide useful information for the development of protocols for assessing driving abilities among novice road users. Indeed, the relation between risk proneness and riding style may allow for the identification of road-user populations who require specific training.

## Introduction

As reported by the European Road Safety Observatory [ERSO] (2006), young drivers (i.e., drivers whose age ranges from 16 to 24 years old) are the most involved category in road accidents, with risk factors from two to three times higher than those of more experienced road users. Road crashes account for 35% of all deaths among this category, representing its primary cause of death (European Road Safety Observatory [ERSO], 2006). The World Health Organization (WHO, 2015) stressed that almost half of road victims are pedestrians, cyclists, and motorcyclists. Indeed, motorcyclists and moped riders are frequently labeled as “vulnerable” road users because of their great physical vulnerability (WHO, 2015; ITF, 2016).

McKenna (2012) suggested that when considering the overrepresentation of young drivers among road crash victims, it is important to take both driving experience and developmental factors into account. Indeed, he showed that the reduction in crash rate is rapid during the 1st months of having a driving license (McKenna, 2012). However, the literature usually distinguishes between on-road experience (i.e., the extent of on-road experience, frequently measured by the number of years since obtaining a riding and/or driving license, as in McKenna) and exposure (Carrol, 1971; Brown, 1982). The latter concept refers to the amount of traffic configurations one faces that may result in a road crash, which is supposed to be proportional to annual mileage. Thus, on-road exposure seems to affect crash rate more than road experience.

Moreover, despite young drivers being more prone to accidents due to their low road exposure, the fact that not all of them have more accidents (Lucidi et al., 2010) indicates that other variables also influence crash rate. Consequently, many efforts have been devoted to identifying the developmental, social, and psychological factors that may be associated with the risk of accidents. Reduction in expert/adult supervision and peer influence are social factors that have been more frequently linked to accident proneness (McKenna, 2012). Regarding psychological aspects, many potential predictors have been studied, but their roles and relationships are still partly unclear. The most important models report the involvement of variables such as antisocial tendencies, impulsivity (McKenna, 2012), aggression, anxiety, normlessness (Ulleberg and Rundmo, 2003), locus of control (Deery and Fildes, 1999; Lucidi et al., 2010; Marengo et al., 2012), emotional adjustment (Deery and Fildes, 1999) and altruism (Marengo et al., 2012). However, sensation seeking has been the most widely studied psychological dimension in this field.

### Sensation Seeking as a Predictor of Risky Driving Behavior

Sensation seeking is “a trait defined by the seeking of varied, novel, complex and intense sensations and experiences and the willingness to take physical, social, legal and financial risks for the sake of such experiences” (Zuckerman, 1994, p. 27). Sensation seeking is considered a multidimensional construct. Indeed, four factors have been identified and are usually assessed on four specific scales: Thrill and Adventure Seeking (TAS), Disinhibition (DIS), Boredom Susceptibility (BS), and Experience Seeking (ES) (Zuckerman et al., 1972). The TAS dimension refers to the attitude toward hazard and speeding in everyday activities; DIS includes behaviors such as gambling, use of alcohol or substances, and high-risk sexual experience (Zuckerman et al., 1972). The BS dimension refers to the aversion to routine and predictable experience, while ES indicates the preference for novelty and for a variety of unconventional experiences (Zuckerman et al., 1972).

Sensation seeking has been studied as a predictor of risky driving—i.e., every behavior that might increase the likelihood of incurring crashes. As Jonah (1997) pointed out, by the end of the 1990s, at least 40 studies had examined this issue, and most found a positive relationship between sensation-seeking level and several factors of risky driving (e.g., drunk driving, speeding, negative attitudes toward the use of seat belts, running red lights, or not stopping at signs). Concerning the role of the single dimensions of sensation seeking in predicting driving behaviors, the TAS subscale seems to be the most involved in risky driving, followed by the DIS and BS scales (Jonah, 1997). Sensation seeking is also associated with increased risk of collisions (Jonah, 1997).

Considering that sensation seeking is not a stable trait, with higher levels shown during adolescence and youth (Zuckerman, 1971), it represents a predictor of reckless driving, especially among young road users (Jonah, 1997; Dahlen et al., 2005; Cestac et al., 2011; Smorti and Guarnieri, 2014). Jonah (1997) proposed two alternative hypotheses to explain this relationship. The first is that the so-called sensation seekers do not correctly perceive the risk of some on-road situations because of their overconfidence in their driving skills. The second explanation is that sensation seekers normally perceive the risk but accept it to experience the thrill (Jonah, 1997; Cestac et al., 2011). In these circumstances, the level of risk perceived by sensation seekers decreases if negative outcomes do not occur, which leads them to engage more frequently in on-road risky behaviors (Jonah, 1997). Because of the prevalence of higher sensation-seeking rates among young males, Deery (1999) suggested that this category may be more likely to accept risks while driving. This makes sensation seeking a variable that deserves consideration when assessing young drivers’ and riders’ behaviors.

### Risky Driving, Sensation Seeking, and Decision Making

So-called “risky drivers” are more prone to show personality features that are connected to risky decision making (Brown et al., 2016). Decision making refers to the set of mental processes underlying many everyday activities. It implies the availability of different options (actions or thoughts) and the need to choose—i.e., to decide which of them is the best one to reach a goal (Beach, 1993). When the outcome of this processing leads one to choose options that have a certain likelihood to result in dangers, we can speak of risky decision making or risk taking. The assessment of this dimension *per se* (outside the context of real life; hereafter non-contextual decision making) is usually made through laboratory tasks such as the Iowa Gambling Task – IGT– among others.

The IGT was developed in order to assess this kind of non-contextual decision making impairment in adults with orbitofrontal damages who showed risk-taking behaviors in everyday life (Buelow and Suhr, 2013). In its standard version, participants have to pick one card at a time from four different decks. Each card is associated with a reward but sometimes also with a loss, the frequency of which varies across decks. Choices from decks A and B (both “disadvantageous” but differing in the frequency at which penalties are delivered) result in an overall net loss after 10 selections. Conversely, decks C and D (termed “advantageous” and with the same difference in loss frequency as in decks A and B) lead to a positive net gain after 10 cards. Normal participants are expected to start choosing randomly from the four decks: Good decision makers are supposed to increase their selections from the advantageous decks as the task continues. Indeed, the tendency to select more cards from the disadvantageous decks (especially in the last trials of the task) is considered a measure of risky decision making (Buelow and Suhr, 2013).

Using this task, Buelow and Suhr (2013) investigated whether a number of predictors such as sensation seeking, impulsivity, and mood are related to deck selections in the IGT, with a particular focus on the last trials of the task, usually considered a measure of decision making under risk. The results showed that high sensation seekers made more disadvantageous choices in the IGT than other participants; that is, they more frequently chose to pick cards from the disadvantageous decks (Linnet et al., 2006; Buelow and Suhr, 2013). Poor performance in the IGT has also been reported among both young adults and adolescents with high DIS in the SSS (Crone et al., 2003): Participants with high DIS scores picked a higher number of cards from the disadvantageous decks than participants with low DIS scores in the last trials of the task.

As stated above, decision making is involved in several everyday activities, including driving. Indeed, drivers have to make a lot of choices about speed, overtaking, trajectory, headway distance, and so on (Deery, 1999). The outcomes of these choices determine the way in which people decide to drive, or their “driving style” (for a review see Sagberg et al., 2015).

Thus, decision making represents an interesting topic in the field of road safety. For instance, a number of studies (French et al., 1993; Farah et al., 2008; Lev et al., 2008; Le Bas et al., 2015; Ba et al., 2016) have tried to investigate the link between non-contextual decision making and the task-specific decision-making skills involved in driving behaviors. Farah et al. (2008) used a driving simulator to assess the association between driving behavior and IGT performance. The results showed that riskier behaviors in the simulator (e.g., greater number of overtaking and higher speed) correlated positively with the number of selections from the disadvantageous decks. On the other hand, mixed findings were reported when risky driving was measured through questionnaires. For instance Ba et al. (2016) found that riskier drivers tended to pick cards from decks in which the penalties were small (although more frequent) in the IGT, independently from the overall net gain, whereas Le Bas et al. (2015) failed in founding relations between IGT performance and on-road risky behaviors. One limitation of the IGT that could explain the inconsistency in the data is the fact that participants’ personality and mood seem to affect their performance in the IGT as well (Buelow and Suhr, 2009). In other words, if non-contextual decision-making skills affect task-specific decision making depending on other personality traits of the participants, then in the studies in which personality traits are not also controlled for, the results may not be clearly interpretable.

Another limitation of the IGT is the fact that IGT performance might also depend on the ability to have conscious access to the rationale of the task (i.e., to consciously identify the “good” and “bad” decks) (Maia and McClelland, 2004) but the majority of studies that have employed the IGT in clinical samples have demonstrated that identifying the differences between the decks does not guarantee optimal decision-making performance in the IGT (Bechara et al., 2005).

In line with the latter consideration, it has been proved that the degree of consciousness about our own decision processes plays a role in driving behaviors as well. Indeed, Malhotra et al. (2017) explored the effect of Decision reinvestment (i.e., the predisposition to consciously monitor and control decision processes) on simulated driving and found that it is negatively correlated with speed choice but positively correlated with bad driving outcomes: Participants who have a higher predisposition to consciously control their decisions drive slower in risky scenarios, but also seem more prone to crashes and infringements.

In summary, relations have been demonstrated between risky driving, sensation seeking, and decision making, but some data inconsistency suggests the need to deeper investigate the way in which decision making and sensation seeking together concur in determining risky driving.

### Virtual Reality and the Assessment of Driving Abilities

As stated in section “Risky driving, Sensation Seeking, and Decision Making,” one way to assess driving abilities to investigate their links with non-contextual decision making is through the use of simulators. The use of virtual reality provides several advantages, especially in road safety research. For example, simulators allow for the study of participants’ behavior in hazardous traffic configurations as well as the collection of performance variables such as speed and acceleration (Farah et al., 2008). Given the possibility of administering the same scenarios to all participants, simulators also allow for full experimental control. The validity and reliability of these tools have been widely discussed (de Winter et al., 2009; Shechtman et al., 2009; Mayhew et al., 2011; Underwood et al., 2011). Overall, the evidence suggests that driving behaviors in simulators are comparable both to self-reported driving behaviors and to on-road performance.

In the last decade, some research has focused on the Honda Riding Trainer (HRT). This is a moped-like simulator that has been specifically designed to train people on safe riding through the administration of risky scenarios based on the Motorcycle Accidents in Depth Study report (MAIDS, 2004). It provides different types of courses (i.e., principal, secondary, and touristic roads) and the possibility to set various options, both for the vehicle (size, type of transmission) and for the environment (night, day, fog). Several studies have demonstrated that the HRT is a valid tool for training novice riders in riding abilities (Vidotto et al., 2008, 2011, 2015; Tagliabue et al., 2013) and enhances their hazard avoidance and risk perception (Tagliabue and Sarlo, 2015; Tagliabue et al., 2017). In addition, the influences of crucial factors such as circadian rhythms, eye movements, mental workload and emotional sounds on driving behavior have been investigated using the HRT as a riding assessment tool (Di Stasi et al., 2009, 2010, 2011; Megias et al., 2011; Del Rio-Bermudez et al., 2014). Moreover, Marengo et al. (2012) compared the performance in the simulator among different risk profiles identified on the basis of personality variables of novice riders. The results showed that the group labeled “at risk” had the worst riding performance in terms of accidents and riding safety. Finally, relations were found between HRT performance on the one hand and dangerous driving and aberrant driving behaviors (as measured by the Dula Dangerous Driving Index and the Driver Behavior Questionnaire) on the other hand (Gianfranchi et al., 2017).

### Aims of the Study

The present study arose from all of these considerations, with the general aim to provide a more integrated framework of the relations among sensation seeking, non-contextual decision making, and driving style by exploring their mutual relations. Moreover, we have thought that the strategy typically used in the literature to investigate the influence of sensation seeking and decision making on driving style has led to consider only one (or a few) index of driving performance (usually crash rate, or speed or an overall score of performance). However, driving style is characterized by several aspects that concur in determining the quality of performance. Thus, we reasoned that the availability of different parameters of driving performances might allow more specific profiling of driving style.

Thus, we decided it could be more interesting and informative to investigate if different riding styles (as measured by the HRT moped riding simulator) were characterized also by different levels of sensation seeking and non-contextual decision making. For this reason, we chose to use the HRT simulator to assess riding abilities in a sample of road users with different degrees of road exposure, so as to identify riding styles with specific behavioral patterns. The first prediction was that different riding styles should be linked to different levels of sensation seeking. In particular, on the basis of the literature, we expected the TAS subscale to be involved.

Then, in the next step, we reasoned that, as reported by Jonah (1997), “sensation seeking may account for only ca. 10–15% of the variance in risky driving” (Jonah, 1997; p. 660). This means that it is not enough to consider just sensation-seeking level when assessing risky driving behaviors. As explained in the section “Introduction,” we considered that among the wide variety of risky driving predictors, non-contextual decision making is one of the most interesting. Indeed, the results regarding the relationship between decision making and risky driving, although sometimes controversial, seem to suggest that worse non-contextual decision-making skills correspond to riskier on-road behaviors (French et al., 1993; Farah et al., 2008; Lev et al., 2008; Le Bas et al., 2015; Ba et al., 2016), assessed either with self-report tools or with driving simulators. On the other hand, there is evidence that sensation seeking may be involved in decision-making tasks, such as the IGT (Crone et al., 2003; Linnet et al., 2006; Buelow and Suhr, 2013). In particular, the TAS subscale seems to play a role in predicting riskier decisions on the IGT (Linnet et al., 2006; Buelow and Suhr, 2013). This evidence and the documented implications of the TAS in determining a risky driving style led us to wonder if a relation between this subscale and the IGT performance was present in our participants, and whether this two dimensions interact in influencing riding behavior. Thus, our second prediction was that at least the TAS score correlated with the IGT score.

Finally, we decided to investigate the combined effects of sensation seeking and non-contextual decision making on riding parameters. Thus, the third prediction was to find modulatory influences of sensation-seeking levels and quality of non-contextual decision making (as measured by the IGT) on risky simulated behaviors.

## Methodology

### Participants and Sample

One hundred and thirty-one students (89 females and 42 males; Mean age: 19.78 years old) at the University of Padua took part in the study. Both license types and mileage were assessed, but 20 participants did not answer the questions regarding these factors. Of the remaining 111 participants, 60 reported a mileage of less than 5,000 km/year, while 51 declared a mileage of more than 5,000 km/year. With regard to license types, 86 had a car driving license (among them, 30 had also a moped riding license), 5 had only a moped riding license (and no other type of license), and 20 did not have any type of license. Overall, 53 participants drove a car, 33 drove both a car and a two-wheeled vehicle (at present, in Italy a car driver’s license allows one to ride motorcycles too), and 5 participants rode only a moped. All of the students were paid 25 euros for their participation and had normal or corrected-to-normal vision.

### Tools

#### The Honda Riding Trainer Simulator (HRT)

**FIGURE 1:**
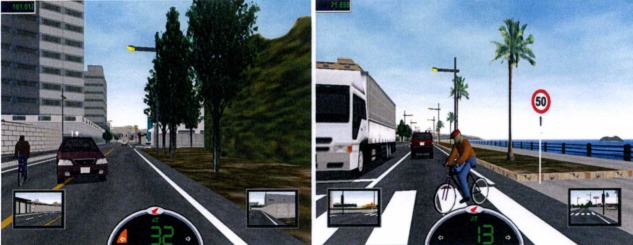
Examples of risky scenes of the Honda Riding Trainer (HRT) simulator.

The HRT equipment resembles a moped and consists of a moped-like seat, handlebars, and an LCD monitor (1024 × 768 resolution) connected through a Pentium 4 PC (Windows XP operating system). Two speakers reproduce typical traffic noise and give instructions on how to use the HRT and on the path to follow during the course. The participants, who were seated approximately 80 cm from the monitor, had to use the handlebars to ride along the virtual courses shown on the monitor, with a visual field covering a horizontal angle of 27.2° and a vertical angle of 21.7°. The simulator was set on automatic transmission mode to prevent any bias from the need to learn how to ride using the foot pedals. The virtual scenarios administered consisted of five courses on secondary roads, with seven or eight risky scenes in each, for a total of 39 scenes (see **Figure 1** for examples of risky scenes from the participant’s perspective).

#### Sensation Seeking Scale V (SSS V)

We used the Italian version of Zuckerman’s Sensation Seeking Scale V (SSS V) to assess the participants’ sensation seeking (Galeazzi et al., 1993; Zuckerman, 1994). It consists of 40 forced choices that investigate various behaviors considered representative of high or low levels of sensation seeking. The questionnaire includes four subscales with 10 items each: Thrill and Adventure Seeking (TAS – with items such as “I would like to try parachute jumping”), Disinhibition (DIS – “I often like to get high, drinking liquor or smoking marijuana”), Boredom Susceptibility (BS – “I get bored seeing the same old faces”) and Experience Seeking (ES – “I like to try new foods that I have never tasted before”). The scores correspond to the sum of the number of high sensation-seeking answers—i.e., a maximum score of 10 for each scale (40 for the overall score). The psychometric properties of the SSS V in both its original and Italian versions have been widely studied over the years. Regarding the validity of the scale, several studies have verified its factor structure (Zuckerman et al., 1978; Galeazzi et al., 1993; Roberti et al., 2006), its convergent-divergent validity (Eysenck and Zuckerman, 1978; Roberti et al., 2006; Zuckerman, 2007), its known-group validity (Zuckerman et al., 1978; Galeazzi et al., 1993; Rossi and Cereatti, 1993), and its criterion validity (Galeazzi and Cavallini, 1994; Roberti, 2004). Regarding the internal consistency of the subscales, the Cronbach’s alpha values for the normative English sample (Zuckerman et al., 1978) were α = 0.81 and α = 0.82 for the TAS in males and females, respectively; α = 0.78 and α = 0.77 for the DIS; α = 0.65 and α = 0.59 for the BS; and α = 0.65 and α = 0.67 for the ES. For the Italian sample Galeazzi et al. (1993) reported the following values: α = 0.80 and α = 0.79 for the TAS in males and females, respectively; α = 0.60 and α = 0.65 for the DIS; α = 0.53 for the BS in both genders; and α = 0.61 and α = 0.69 for the ES.

#### The Iowa Gambling Task (IGT)

We administered a computerized version of the Iowa Gambling Task (IGT) based on the original version conceived by Bechara et al. (1994). The participants started with an imaginary monetary amount of 2,000 euros and were told that their goal was to maximize their profit. They were asked to pick one card at a time from one of the four decks represented on the screen (1920 × 1080 resolution) of an Intel Core i3 PC (Windows 7 operating system): Each card had a monetary win, but some cards could also have a loss. There were no time limits, but the total number of choices was set to 100. The decks were either disadvantageous (decks A and B) or advantageous (decks C and D) on the basis of the amount and frequency of rewards and punishments. The disadvantageous decks led to a sure loss in the long run, whereas the advantageous ones led to a gain. The participants were not informed of the total number of choices they had to make or of the differences between the decks.

Previous studies (Bechara, 2004) have demonstrated that normal control subjects avoid the disadvantageous decks and, in the long run, prefer the advantageous ones, so as to maximize their profit. Participants who are considered bad decision makers or prone to risk-taking behaviors (e.g., patients with orbitofrontal damages, gamblers, substance abusers, high sensation seekers) show the tendency to pick more cards from the bad decks (Bechara, 2004; Bechara and Martin, 2004; Glicksohn and Zilberman, 2010; Buelow and Suhr, 2013). Thus, a greater number of choices from decks A and B are usually associated to worse decision-making abilities in ambiguous or risky contexts.

### Procedure

All of the participants filled in an informed consent form and were told about all of the study procedures before the beginning of the experiment, which consisted of two sessions scheduled a few days apart from each other. Each participant filled in the SSS V and a questionnaire regarding driving and riding experience and road exposure. Then, the sample was randomly split into two groups to counterbalance the order of the tasks. One group (*N* = 66) rode the HRT during the first session of the experiment and performed the IGT during the second session, while the other group (*N* = 65) performed the IGT during the first session and rode the simulator in the second session.

The task with the HRT simulator consisted of five test courses on secondary roads preceded by one practice course to familiarize the participants with the simulator equipment. At the beginning of the task, the participants were instructed on how to ride the simulator. The experimenter explained how to start the engine and how to use the throttle, the brakes, and all the controls. The transmission was set to automatic so as to prevent any bias linked to unfamiliarity with the foot controls. The familiarization course was the same for each participant: It lasted for about 3 min, during which the participants explored the virtual environment without other vehicles. Meanwhile, the experimenter monitored participants’ behavior, answering any further questions on how to use the HRT at the end of the familiarization course. After that, the five test courses began, and the participants were told to ride along the courses as if they were on the road, respecting the traffic rules and avoiding collisions. They also had to follow the instructions provided through the speakers that indicated the pathway similarly to the directions provided by satellite navigators. For example, some seconds before participants approached a crossing road, the speakers gave the instruction “Turn right at the next intersection.”

At the end of each course, the system provided a score that informed participants about their performance: Worse evaluations corresponded to more hazardous performance. Afterward, there was a rest period of about 2 min to prevent fatigue. Overall, the task on the simulator lasted for about 45 min.

None of the participants had previous experience with training in a riding/driving simulator, with the SSS, or with the IGT. The study was approved by the Ethics Committee for Psychological Research of the Departments of Psychology, University of Padua.

### Coding

We monitored the participants’ behaviors on the HRT to assess their riding performance. In particular, we used the variables recorded in the .csv output files of the simulator to deeply assess each participant’s performance. Thus, we extracted 18 indexes for each of the five courses. Specifically, the indexes were: The means and standard deviations of the throttle opening (in percentages, as an indirect indicator of the acceleration behavior), pressure on the front and rear brakes (Kg), speed (Km/h), and on-road instability (measured as horizontal deviations from the right side of the roadway), as well as the number of brakings, accidents, and path sections where participants’ speed exceeded the limit. Moreover, we also extracted the amount of time spent over the speed limit (i.e., the sum of the frames over the speed limit), the mean and the maximum values of over-speeding, and a summary value, labeled “Evaluation score,” which corresponds to the mean of the scores that the HRT automatically gives for every performance. A higher score represents a worse and riskier performance.

With regard to the IGT, the participants were expected to start choosing randomly from the four decks or from the disadvantageous ones, slowly shifting their choices toward the advantageous decks as the task progressed. Following the literature, we divided the task into 5 blocks of 20 trials each to assess the participants’ behavioral changes during the IGT (Bechara et al., 1999, 2000, 2001; Bechara and Martin, 2004). For each block, we first calculated one of the most frequently used measures of IGT performance (Bechara et al., 1999, 2001; Bechara and Martin, 2004)—the Net Score within each block, obtained by subtracting the sum of the disadvantageous selections from the sum of the advantageous ones. A higher score corresponds to a higher number of picks from the advantageous decks—i.e., better decision making. Then, as an overall measure of performance in the IGT, we computed the Goodness of Decision-Making (GDM) score. It was calculated as the mean Net Score obtained by participants in the third, fourth, and fifth blocks, since, as shown by Bechara and Martin (2004) and pointed out by Buelow and Suhr (2013), the last 40–60 choices refer to decision making under risk, as the participant has already had the opportunity to learn the costs and benefits of the different decks. Thus, we identified as good decision makers the participants who reached GDM scores higher than the median of the sample, whereas the participants who got lower GDM scores than the median of the sample were labeled as bad decision makers (Linnet et al., 2006; Glicksohn and Zilberman, 2010).

For the SSS V, the original scoring instructions were followed. The maximum total score was 40, and each subscale (TAS, DIS, BS, and ES) had a maximum score of 10.

## Analyses And Results

Overall, the statistical analyses can be divided into two steps. The first one dealt with identifying the riding profiles through a cluster analysis. The second one focused on the relationships among these risk profiles, sensation seeking, and decision making.

Concerning this second step, we performed this comparison in two directions: In line with our first prediction we tested differences in sensation seeking and non-contextual decision making as depending on the different riding styles identified. Thus, we carried out a MANOVA on the dependent variables SSS V scores and GDM score since, compared with simple ANOVA, Multivariate ANOVA takes into account the inter-correlations among the dependent variables and reduce the experiment-wise level of Type I error.

Then, on the basis of the literature which showed evidence of relations between some subscales of SSS and IGT performance, Spearman correlations were calculated between GDM and SSS scores to investigate whether the same relations are evident also in our participants (second prediction).

Finally, to test our third prediction, we carried out a second MANOVA focused on the scores significantly correlated, to identify conjoint influence of sensation seeking and non-contextual decision making on all the parameters of the riding performance. As before, we reasoned that Multivariate ANOVA would allow to reduce the experiment-wise level of Type I error by taking into account the inter-correlations among the dependent variables (riding performance parameters).

All of the analyses were conducted using the IBM SPSS 23 statistical package.

### Cluster Analysis and the Cluster Solution

In order to identify the riding profiles, a cluster analysis was run using the 18 HRT indexes as grouping variables (see the Supplementary Material for the mean and standard deviation of each HRT index). As recommended by the literature (Lucidi et al., 2010; Marengo et al., 2012), we used Ward’s method of hierarchical clustering with squared Euclidean distance measures as the first step. All of the grouping variables were standardized (into *Z*-scores). Three clusters emerged upon inspection of the dendrogram and the merging coefficients. Thus, we used the *K*-means clustering method with the centroids of the previous Ward’s analysis to reach the most appropriate definitions for the groups. The final solution identified three clusters with different riding patterns. The first one, named “Prudent,” was composed of 45 participants (31 females and 14 males, 69 and 31%, respectively), with a mean age of 19.45 years old. Among them, 23 had an annual mileage of less than 5,000 km, and 17 had a mileage of more than 5,000 km. The second cluster (“Imprudent”) was composed of 29 students (16 females and 13 males, 55 and 45%, respectively), with a mean age of 20.07 years old. Twelve of them declared an annual mileage of less than 5,000 km, while 9 declared more than 5,000 km. The third cluster, labeled “Insecure,” included 57 participants (42 females and 15 males, 74 and 26%, respectively). The mean age was 19.9 years old; 25 students declared less than 5,000 km/year, and 25 reported more than 5,000 km/year. Among the participants who drove a car, 21 (40%) fell in the Prudent cluster, 10 (19%) in the Imprudent cluster, and 22 (41%) in the Insecure cluster. Among the participants who rode a two-wheeled vehicle, 16 (42%) fell in the Prudent cluster, 6 (16%) in the Imprudent cluster, and 16 (42%) in the Insecure cluster.

**FIGURE 2:**
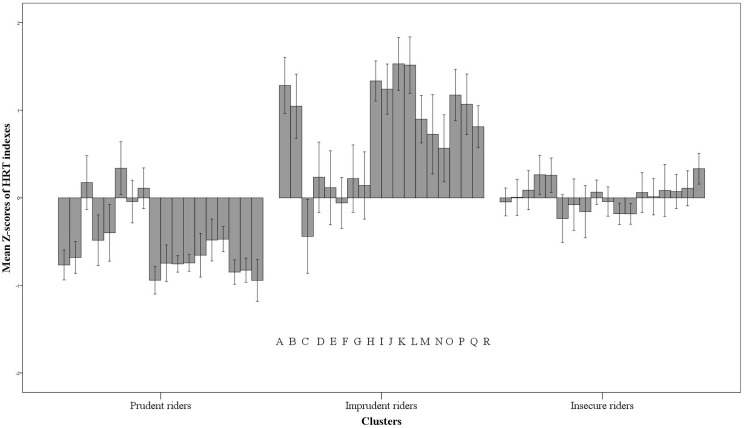
Trends of the three clusters on the 18 HRT indexes. The indexes are reported for all three clusters in the order displayed by the letters on the bottom of the panel. The indexes are: Mean of the throttle opening (A) and its Standard deviation (B), Number of breakings with the front brake (C), Mean (D), and Standard deviation (E) of front brake pressure, Number of brakings with the rear brake (F), Mean (G), and Standard deviation (H) of rear brake pressure, Mean (I) and Standard deviation (J) of speed, Time spent over the speed limit (K), Number (L), Mean (M), and the highest value (N) of overspeeding, Mean (O) and Standard Deviation (P) of on-road instability, Number of accidents (Q) and Evaluation score (R). Vertical bars represent standard errors.

Although we have no information about road exposure for 20 of the participants, a chi-square test confirmed that there were no significant differences between the clusters in terms of annual mileage [χ^2^(2) = 0.603, *p* = 0.740]. As reported in **Figure 2**, each cluster showed a peculiar trend in HRT performance, with a clear opposite trend between the first and second clusters, while the third can be described as intermediate.

As can be seen in **Figure 2** (which shows the mean *Z*-scores of the HRT indexes for the clusters), the second cluster is characterized by the highest values in almost all of the indexes, indicating less safe behavior (e.g., higher speed and acceleration rate, higher number of accidents, and worse performance). The other two clusters can be labeled as “Prudent riders” and “Insecure riders.” Indeed, the first group showed a peculiar riding style with, for instance, very low values for speed, acceleration, and accidents and the best (i.e., the lowest) Evaluation score. On the other hand, the Insecure group seemed to be characterized by a behavioral pattern that can be described as intermediate between the other clusters. These participants may represent the average rider, who does not ride recklessly but whose behavior is not as safe as possible. In addition, they showed some elements of insecurity, such as a trend of braking suddenly, even though their speed was lower compared with that of the Imprudent participants.

### Riding Styles, Sensation Seeking, and Decision Making

After identifying the risk profiles, the next step was to compare the HRT performance with the scores on the SSS V and on the IGT. Thus, we ran the first MANOVA on the four subscales of the SSS V and on the GDM score, with three between factors: *Group* (i.e., the clusters; 3 levels) ×*Gender* (2 levels) ×*Road Exposure* (i.e., above or below the cutoff value of 5,000 km/year; 2 levels). The analysis was run on 111 subjects because of the missing data regarding the annual mileage.

Factorial MANOVA revealed significant multivariate effects for the factor *Gender*, Wilks’ aaa=0.84, *F*(5,95) = 3.64, *p* < 0.01 and for the interaction *Group* ×*Gender*, Wilks’ aaa = 0.82, *F*(10,190) = 2.02, *p* < 0.05. Univariate results indicated that *Gender* reached significance for the BS subscale, with *F*(1,99) = 8.76, *p* < 0.01, *MSe* = 3.17: Males showed higher BS scores (3.96 vs. 2.81) than females.

Moreover, at the univariate level, the interaction *Group* ×*Gender* reached significance for the TAS subscale with *F*(2,99) = 4.45, *p* < 0.05, *MSe* = 6.74 and for the DIS subscale with *F*(2,99) = 4.91, *p* < 0.01, *MSe* = 3.24. Insecure males showed lower TAS scores than Insecure females (4.60 vs. 6.70; *p* < 0.05 at the *post hoc* comparisons) and Prudent males (7.90; *p* < 0.01). On the other hand, Prudent males showed higher DIS scores than Prudent females (5.87 vs. 4.26, *p* < 0.05), and Prudent females showed lower DIS scores than Insecure females (4.26 vs. 5.60; *p* < 0.05).

In order to investigate the presence of relations among the risk profiles, sensation seeking and performance in the IGT, we first found the Spearman correlations between the SSS V subscale scores and the score of GDM calculated on the basis of performance in the IGT task. Confirming data from the literature regarding the relations among sensation seeking, thrill seeking, and risk taking (Zuckerman et al., 1972; Linnet et al., 2006; Glicksohn and Zilberman, 2010), the TAS scores in our sample showed a negative correlation with IGT performance, as measured by the GDM score (*r* = -0.24, *p* < 0.01). Since, as it has been previously explained, higher GDM indicates better performance, the negative correlation suggests that high thrill and adventure seekers also showed worse non-contextual decision-making ability (**Table 1**).

**Table 1:** Spearman coefficients of correlation between Sensation Seeking Scale V (SSS V) and Goodness of Decision-Making (GDM) scores.

SSS V	GDM score	*p*-values
TAS	-0.24	0.006
ES	0.09	0.282
DIS	-0.03	0.696
BS	-0.03	0.702
Total score	-0.09	0.284

Then, on the basis of the TAS scores, we identified high and low thrill and adventure seekers as the participants who got lower or higher TAS scores, respectively, in comparison with the median of our sample (following a procedure already reported in the literature; see Jonah et al., 2001; Rosenbloom, 2003). Analogously, concerning IGT task performance, we labeled the participants who got scores greater than the median of the sample as good decision makers and the participants who got scores less than the median of our sample as bad decision makers (Linnet et al., 2006; Glicksohn and Zilberman, 2010). Thus, in order to explore the combined influence of sensation seeking and non-contextual decision making on driving style, we carried out a MANOVA on all 18 simulator variables with two between-participant factors: *TAS* (2 levels) ×*GDM* (2 levels).

**FIGURE 3:**
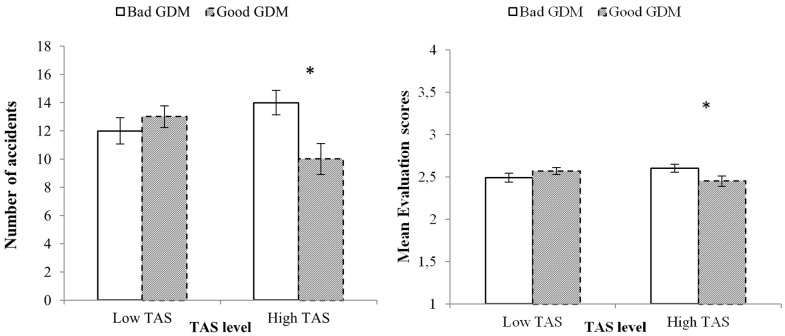
Two-way interaction showing the participants’ Number of accidents (left panel) and Evaluation scores (right panel) for their moped riding performance. The asterisks indicate significant *post hoc* pairwise comparisons (*p* < 0.05) with Bonferroni correction. The Evaluation scores ranged from 1 (better performance) to 4 (accidents), so that higher scores indicated worse performance. Vertical bars represent standard errors.

Factorial MANOVA revealed a significant multivariate effect for the interaction *TAS* ×*GDM*, Wilks’ aaa = 0.76, *F*(18,110) = 1.89, *p* < 0.05. Univariate results indicated that the interaction *TAS* ×*GDM* was significant for Number of accidents and mean Evaluation scores in the simulator, with *F*(1,127) = 4.06, *p* < 0.05, *MSe* = 26.45 and *F*(1,127) = 5.03, *p* < 0.05, *MSe* = 0.078, respectively. High thrill and adventure seekers who were also bad decision makers had significantly more accidents (14 vs. 10) and showed worse riding simulator performance (2.60 vs. 2.45) than high thrill and adventure seekers who were good decision makers (**Figure 3**).

## Discussion

Following an innovative approach to the issue of evaluating driving abilities among young road users, the present study investigated the possibility of employing the HRT simulator as a tool to assess riding abilities among young participants with different degrees of road exposure. A cluster analysis was run on the riding indexes collected by the HRT, identifying three groups corresponding to different riding profiles: Prudent, Imprudent, and Insecure riders. Furthermore, in line with our first prediction, differences emerged among the clusters in terms of sensation seeking and gender; that is, Insecure and Prudent groups show modulation by gender on TAS and DIS dimensions. Concerning our second prediction, links between sensation seeking and non-contextual decision making were also confirmed, as attested by the significant correlation between TAS and GDM scores. Finally, the third prediction was confirmed too, in that results show that the effect of sensation seeking on driving performance is modulated by non-contextual decision making.

As Zuckerman et al. (1980) stated, gender differences in sensation seeking (with males usually scoring higher on the TAS and DIS subscales and the total SSS score) seem to be related both to differences in social experience between genders and to different levels of monoamine oxidase (which seems to modulate the sensitivity of the brain reward centers through dopamine) in males and females (Zuckerman et al., 1980). It is worth noting that our data only partially confirm previous results, showing higher DIS among males (Zuckerman, 1971; Zuckerman et al., 1972), but only in the Prudent group. On the contrary, in the Insecure group, females are more TAS than males. More interestingly, the fact that Prudent females are less disinhibited than Insecure females seems to indicate that DIS has a detrimental effect on riding performance in females, whereas the fact that Prudent males are more TAS than Insecure males might suggest that TAS does not necessarily represent a disadvantageous trait, at least in males. Taken together, all these results confirm that gender differences need to be taken into account when assessing the influence of personality traits on driving abilities, because they might affect every-day life (such as driving) differently in males and females. Moreover, they indicate that sensation seeking *per se* does not necessarily lead to risky driving behaviors.

Overall, the results of our study show that bad decision makers with higher TAS scores are more prone to accidents and have generally worse riding performance (i.e., they are less safe) on the HRT. In other words, high thrill and adventure seekers are also risky riders only if they are simultaneously bad decision makers in the IGT. Note that Jonah (1997) explained the proneness of sensation seekers to risky driving behaviors with two possible alternatives. High sensation seekers may perceive less risk in a variety of on-road situations, or they may have a correct risk perception but accept the risk so as to experience the thrill of that situation (Jonah, 1997) and to maintain their level of arousal (Zuckerman et al., 1972). The present result is in favor of a further alternative explanation: high sensation seekers with optimal non-contextual decision-making skills do not drive dangerously, whereas high sensation seekers with poor performance in a non-contextual decision-making task do. Different from Jonah’s explanations, our finding suggests that general decision-making skills and sensation seeking are independent factors that contribute jointly in determining driving performance.

There is another alternative explanation of the influence of personality traits and decision making on risky driving ability—i.e., Decision reinvestment (Malhotra et al., 2017), defined as the propensity to control our decision processes in a conscious way. This factor has been proved to affect driving behavior in simulated risky scenes (Malhotra et al., 2017) in which participants with high Decision reinvestment attitude drove more slowly in risky situations, but were also more prone to involvement in crashes. Thus, Decision reinvestment might represent a variable that modulates decision making and consequent behavior in the driving context. However, the apparently counterintuitive result of a lower speed associated to a greater likelihood of being involved in crashes represents an indirect confirmation of the usefulness of the approach suggested in the present work to profile driving performance on the basis of a large number of indexes (not just one or a few) since each parameter (for instance the speed) does not necessarily provide enough information about the degree of riskiness of the driver’s performance. Despite the fact that we did not consider the propensity to control consciously decision processes in our participants, the results of Malhotra et al. (2017) support the idea to take into account a range of parameters when assessing risky driving behaviors.

Besides these results, the present data represent the first confirmation of the possibility of employing the HRT simulator as a tool for assessing riding abilities among young road users with different degrees of road exposure. The clusters identified here are comparable to those reported by Lucidi et al. (2010) and Marengo et al. (2012). Indeed, both studies reported the presence of three groups of young drivers in their samples, clustered on the basis of psychological variables. In particular, Marengo et al. (2012) named the clusters “Risky,” “Worried,” and “Safe” drivers, respectively. The three groups showed different behavioral patterns in the HRT in terms of accidents and performance safety. Our study, partially replicating these findings, followed a different approach, in that we clustered the participants on the basis of their riding performance. In addition, the riding assessment was conducted on a wide number of variables, which allowed for a deep inspection of the participants’ behavior. To the best of our knowledge, this represents an innovation in the field of clustering research for driving assessments, which are usually based on self-reported behaviors (Ulleberg and Rundmo, 2003; Lucidi et al., 2010; Marengo et al., 2012).

This approach to assessing driving abilities has some important advantages. First, the use of a riding simulator allows for the identification of risky profiles on the basis of behavioral data that overcome the limits of self-report tools (e.g., social desirability bias). Second, virtual reality offers the opportunity to test driving abilities and behaviors in a safe environment. This also involves the chance to assess these abilities in samples with poor or even no on-road experience. Finally, riding/driving simulators can collect a huge amount of data regarding a wide number of parameters (e.g., acceleration, lateral position, stability), allowing for a detailed monitoring that would not be possible without them. Thus, the development of integrated assessment protocols, such as the use of virtual reality, self-report tools, and decision-making tasks, may represent an opportunity to identify, with a good degree of accuracy, road users with specific characteristics who may be more at risk while riding/driving and who may need more focused training.

Some limitations and future perspectives of this study need to be mentioned. First of all, while on the one hand, the fact that the three driving styles do not differ for annual mileage might suggest that driving style is a relatively independent and stable behavior modality, on the other hand, this may seem counterintuitive, considering that practice has been demonstrated to improve the mechanisms underlying tasks, so as to lead to better (expert) performance (in line with the deliberate practice framework; Williams and Ericsson, 2005). However, practice develops over the years, during which road users are supposed to collect more and more experience so as to learn to cope with a great variety of risky situations. Thus, the lack of the effect of exposure on riding style in our sample could be due to the young age of our participants. Moreover, it must be taken into account that 20 participants did not provide information regarding their road exposure; consequently, this conclusion is not based on the full sample. Considering the importance of this aspect, future research should investigate whether and how exposure and practice (or experience) interact in determining driving style.

Another limitation of the present work is that the IGT task enables assessment of risky non-contextual decision making in relatively simple laboratory settings, and a number of studies have already succeeded in showing the transferability of this general skill to complex, task-specific contexts such as driving (Farah et al., 2008; Lev et al., 2008; Ba et al., 2016). However, the limitation of this approach relies on the difficulty of considering and assessing the large number of variables potentially involved in complex real-life tasks. The effects of this limitation might be mitigated by an integrated assessment approach such as that proposed in the present study.

Moreover, one might also wonder whether the relation of both sensation seeking and decision making with driving styles assessed through a simulator really transfers to the real world. Several studies have found a link between these dimensions and real on-road behaviors. For instance, Jonah (1997) cited a number of papers that reported correlations between high levels of sensation seeking and both self-reported and recorded crash rate or violations. With regard to decision making, its role in characterizing driving style (for a review see Sagberg et al., 2015) and in allowing discrimination between risky and safe drivers assessed either through self-reported or through recorded on-road violations (Lev et al., 2008; Ba et al., 2016) has already been proved. Thus, on the basis of the literature, it is possible to predict that the implications of both sensation seeking and decision making in driving behaviors as revealed in the present work might be replicable also when considering real on-road context.

Two further limitations concern the familiarity of the participants with the simulator task, in terms of familiarity with virtual driving learning systems or video games, and in terms of familiarity with the moped riding task. Concerning the former, our participants had not had previous experience with other computerized driving learning tasks, but we did not check for experience with video games. Nevertheless, the task on the simulator is different from the most common video games in that it does not reward risky behaviors (the score provided at the end of each course acted as feedback to discourage the competitive behaviors typically observed in video games). Moreover, the presence of the experimenter, along with the request to ride so as to avoid collisions, and the absence of rewards for risk-taking behaviors should have prevented these effects. However, this aspect needs to be considered in future research. Concerning the familiarity with the moped riding task on the road, it can be argued that driving a car is a very different task than riding a two-wheeled vehicle, and this could have affected simulator performance, especially with regard to risk taking behavior. It is worth noting that the distribution of participants who had only on-road car-driving experience in the three groups is very similar to that of the participants who rode two-wheeled vehicles. The fact that the kind of powered vehicle used did not influence the inclusion of participants in the different clusters indicates that the familiarization phase of the present research and the choice of the automatic transmission option should have limited the influence of differences in motor skills (linked to driving or riding habits) and was effective in making homogeneous the starting level when participants approached the actual test phase.

The sample of the study, although generally adequate, prevents us from drawing firm conclusions regarding gender differences because of the prevalence of females in the sample. Therefore, further studies should also investigate the presence of gender differences. Again, the simulator assessment was conducted on five courses. For a complete assessment, more courses with different scenarios and characteristics (e.g., fog, night, principal and secondary roads) could be employed.

## Conclusion

Besides the already discussed limitations, the present study, in line with other previous evidence (Lucidi et al., 2010; Marengo et al., 2012), suggests that young road users are not a homogeneous category in terms of riding style when assessed through a riding simulator. Moreover, the quality of decision making seems able to modulate performance in terms of crashes and safety in participants with high levels of thrill seeking. The interaction between these two factors suggests that a multidimensional assessment may be a useful approach for identifying more at-risk groups. Finally, these results represent a further contribution to the attempt to verify whether the HRT simulator can be employed as an assessment tool for riding styles. Taken together, these findings may have important implications for the development of both assessment and training protocols, suggesting that on-road behaviors are the result of several cognitive and psychological factors and encouraging further in-depth studies on their role and mutual relations.

The present results also suggest interesting implications for older adult drivers. Indeed, to date, the majority of studies have focused on the decline in driving abilities among older adults as a consequence of general cognitive decline (Schwebel et al., 2007). Moreover, in a sample of drivers more than 75 years old, Schwebel et al. (2007) found that higher sensation-seeking levels correlated with a higher number of violations and tickets. However, when considering the IGT task, older healthy adults perform comparably to young healthy participants and also show better insight on the wins and losses for each deck (Wood et al., 2005). Thus, on the basis of our data, it is possible to expect that, despite cognitive decline, any improvement in non-contextual decision-making skills might represent a protective factor in older drivers too.

Of course, when virtual reality is used to assess driving behaviors, the replicability of results in the real world is a crucial matter. Thus, the main future research direction should be a follow-up study aimed at monitoring participants who were previously profiled through an integrated protocol (such as that proposed in the present work) to verify whether the identified profiles are predictive of different levels of risk in the real world. Moreover, comparing groups of participants of different ages might help to shed light on the development of riding/driving styles across the lifespan.

## Author Contributions

MT supervised data collection and contributed to statistical analyses and manuscript writing. EG conducted data collection, statistical analyses, and manuscript writing. AS contributed to data analysis, particularly focusing on the methodological aspects, and manuscript writing. MT, EG, AS, and GV contributed to research planning and results discussion.

## Conflict of Interest Statement

The authors declare that the research was conducted in the absence of any commercial or financial relationships that could be construed as a potential conflict of interest.
