# Supplementary material for: Sensation Seeking, Non-contextual Decision Making, and Driving Abilities As Measured through a Moped Simulator
Source: Front Psychol. 2017 Dec 11;8:2126. doi: 10.3389/fpsyg.2017.02126 (PMC5732172; doi:10.3389/fpsyg.2017.02126)
Supplement: Supplementary file 1 [file Supplementary_Material.docx]

| **HRT indexes** | **Mean** | **Standard deviation (SD)** |
| --- | --- | --- |
| Throttle opening (%): Mean | 24.40 | 7.34 |
| Throttle opening (%): SD | 20.43 | 5.75 |
| Brakings with the front brake: Number | 113.82 | 43.41 |
| Brakings with the front brake (Kg): Mean of pressure | 10.39 | 3.25 |
| Brakings with the front brake (Kg): SD of pressure | 7.35 | 1.77 |
| Brakings with the rear brake: Number | 86.24 | 67.89 |
| Brakings with the rear brake (Kg): Mean of pressure | 5.78 | 3.64 |
| Brakings with the rear brake (Kg): SD of pressure | 3.74 | 2.06 |
| Speed (Km/h): Mean | 18.67 | 2.80 |
| Speed (Km/h): SD | 8.27 | 1.00 |
| Time spent over the speed limit (Frames) | 939.99 | 847.84 |
| Overspeeding: Number | 10.74 | 9.27 |
| Overspeeding (Km/h): Mean | 2.25 | 1.20 |
| Overspeeding (Km/h): Highest value | 9.91 | 5.94 |
| On-road instability (m): Mean | .0015 | .0009 |
| On-road instability (m): SD | .016 | .004 |
| Accidents: Number | 12.92 | 5.21 |
| Evaluation score (range: 1-4): Mean | 2.54 | .28 |

**APPENDIX**

Mean and standard deviation (SD) of each HRT index
